# Opposite Effects of HIV-1 p17 Variants on PTEN Activation and Cell Growth in B Cells

**DOI:** 10.1371/journal.pone.0017831

**Published:** 2011-03-14

**Authors:** Cinzia Giagulli, Stefania Marsico, Anna K. Magiera, Rosalinda Bruno, Francesca Caccuri, Ines Barone, Simona Fiorentini, Sebastiano Andò, Arnaldo Caruso

**Affiliations:** 1 Department of Experimental and Applied Medicine, University of Brescia, Brescia, Italy; 2 Department of Pharmaco-Biology, University of Calabria, Arcavacata di Rende (Cosenza), Italy; 3 Medestea Research, Turin, Italy; 4 Department of Cell Biology, University of Calabria, Arcavacata di Rende (Cosenza), Italy; Karolinska Institutet, Sweden

## Abstract

The HIV-1 matrix protein p17 is a structural protein that can act in the extracellular environment to deregulate several functions of immune cells, through the interaction of its NH_2_-terminal region with a cellular surface receptor (p17R). The intracellular events triggered by p17/p17R interaction have been not completely characterized yet. In this study we analyze the signal transduction pathways induced by p17/p17R interaction and show that in Raji cells, a human B cell line stably expressing p17R on its surface, p17 induces a transient activation of the transcriptional factor AP-1. Moreover, it was found to upregulate pERK1/2 and downregulate pAkt, which are the major intracellular signalling components involved in AP-1 activation. These effects are mediated by the COOH-terminal region of p17, which displays the capability of keeping PTEN, a phosphatase that regulates the PI3K/Akt pathway, in an active state through the serin/threonin (Ser/Thr) kinase ROCK. Indeed, the COOH-terminal truncated form of p17 (p17Δ36) induced activation of the PI3K/Akt pathway by maintaining PTEN in an inactive phosphorylated form. Interestingly, we show that among different p17s, a variant derived from a Ugandan HIV-1 strain, named S75X, triggers an activation of PI3K/Akt signalling pathway, and leads to an increased B cell proliferation and malignant transformation. In summary, this study shows the role of the COOH-terminal region in modulating the p17 signalling pathways so highlighting the complexity of p17 binding to and signalling through its receptor(s). Moreover, it provides the first evidence on the presence of a p17 natural variant mimicking the p17Δ36-induced signalling in B cells and displaying the capacity of promoting B cell growth and tumorigenesis.

## Introduction

The three-dimensional structure of HIV-1 matrix protein p17, a 132 amino acid (aa) structural protein, has been determined by nuclear magnetic resonance and X-ray crystallography. Individual folded p17 molecules result composed of five major α-helixes and a highly basic platform consisting of three β strands [1], [2]. This partially globular protein presents four helixes centrally organized to form a compact globular domain capped by the β-sheet. Basic residues exposed from the β strands, generally conserved among different HIV-1 strains, are implicated in cell membrane binding [1]. The fifth helix (H5) in the COOH-terminus of the protein, projects away from the packed bundle of helixes to expose COOH-terminal residues essential for early steps during the HIV-1 infectious cycle. The most distinguishing feature when comparing X-ray and NMR solved conformation of p17 is the folding of H5 and results obtained from Verli et al. [3] suggest that the biological form of this protein may have its COOH-terminal portion partially unfolded.

Converging evidences suggest that p17 is generated along all the virus life cycle and plays a critical role in viral replication [4], [5], [6], [7]. *In vitro* experiments have shown that p17 is released by infected cells into the extracellular space [8] and this may occur via alternative secretion pathways [9], [10] or exocytotic pathways [11]. However, the release of p17 in the HIV-infected microenvironment through mechanisms of virus disintegration or lysis of infected cells cannot be ruled out. P17 is detected at nanomolar concentrations in the plasma of HIV-1-seropositive individuals [12] and in several anatomical compartments such as lymph nodes [13] and brain [14] of patients naïve for or successfully treated with highly active anti-retroviral therapy (HAART). Recent reports have also shown that HIV-1 transcription is efficiently induced by different stimuli [15] even in the presence of protease inhibitors [16], providing the evidence that p17 is continuously synthesized and released even under HAART.

Besides its well established role in the virus life cycle, increasing evidences suggest a role for exogenous p17 in deregulating the biological activity of different immune cells, which may be relevant in the context of viral pathogenesis. Indeed, p17 is able of influencing the activation, the differentiation status and the proliferative capacity of different target immune cells as T cells [17], [18], NK cells [19], monocytes [20] and plasmacytoid dendritic cells [12]. Functional activities of p17 depend on the expression of a specific receptor for p17 (p17R) on the surface of different immune cells and on the activation of specific signalling pathways triggered by interaction between the NH_2_-terminal region of p17 and p17R [18]. In particular, experiments performed on primary human monocytes have shown that p17 selectively activates the transcriptional factor AP-1 and triggers these cells to produce monocyte chemotactic protein-1 (MCP-1) [20].

The viral protein shows tendency to oligomerize, forming trimers of different crystal forms [21]. However, this occurs just at high millimolar concentration, as in the intracellular compartment, during viral assembly in the proximity of cell surface. At nanomolar concentration, as in the blood of HIV-1-infected individuals [12], the p17 is present in monomeric form [21]. In this study, we investigate the biological activity of monomeric p17 protein derived from the HIV-1 BH10 (clade B) on Raji cells, a human B cell line stably expressing p17R on cell surface. Here we demonstrate that in these cells p17 transiently activates the cellular transcriptional factor AP-1 by downregulating the Akt pathway, through activation of PTEN (phosphatase and tensin homolog deleted on chromosome 10), a phosphatase which antagonizes PI3K activity. Activation of PTEN was found to be triggered by the Ser/Thr kinase ROCK, a downstream effector of RhoA, already known to control PTEN activity [22], [23], [24]. All these effects were found to be mediated by the H5 in the p17 COOH-terminus, and by the cooperation of at least two distinct functional epitopes on the viral protein. Activation of PTEN and inhibition of Akt are important events in regulating cell cycle progression and proliferation [25] and are responsible for the antiproliferative effect exerted by p17 on B cells. On the contrary, a p17 variant derived from a Ugandan HIV-1 strain, named S75X, was found to trigger opposite signalling pathways, which induce B cell growth and malignant transformation. This finding suggests that p17 derived from divergent HIV-1 strains may exert a distinct biological activity on B cells and possess a different pathogenetic potential.

## Results

### HIV-1 p17 oligomerization in solution is dependent on ionic strength

**Figure 1:**
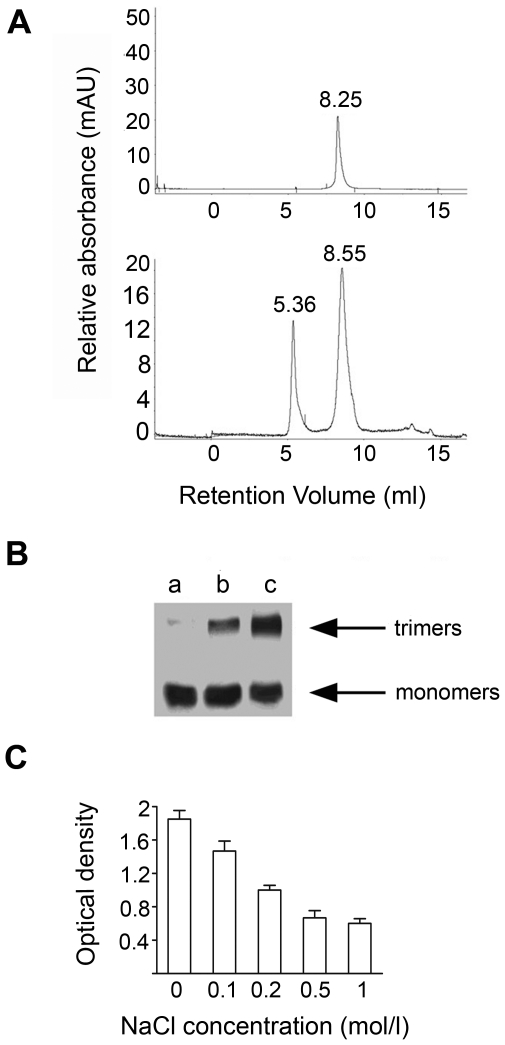
HIV-1 p17 oligomerization in solution depends on ionic strength. (A) Analysis of p17 by gel-filtration under different NaCl concentration. P17 is present in monomeric form from 0.5 to 1 M of NaCl with retention volume of the p17 monomer equal to 8.25 ml (upper panel). Lowering the ionic strength of the solution by dialysis against PBS (0.13 M), we observed a gradual process of p17 oligomers formation, with a volume of retention in gel-filtration of 5.36 ml (lower panel). The panels are representative of four independent experiments with similar results. (B) Western blot analysis of p17 under different NaCl concentration (0.5, 0.2 and 0.1 M). In western blot analysis both monomeric and trimeric p17 are recognized by mAb MBS-3. The detection of an approximately 40.000 Dalton band (trimeric p17) increases with the decreasing of NaCl concentration (lane A: p17 in NaCl 0.5 M; lane B: p17 in NaCl 0.2 M; lane C: p17 in NaCl 0.1 M), while p17 is mostly in a monomeric form in a solution containing 0.5 M NaCl. The blot is representative of four different experiments with similar results. (C) Solid-phase ELISA to evaluate p17 oligomers formation under different saline concentration. The p17 polymers formation evaluated as binding of GST-p17 to p17, detected by mAb anti-GST, decreased by increasing NaCl concentration in the solution. Bars represent the mean ± SD of triplicate samples. These results are representative of four independent experiments with similar results.

In order to simulate the conditions present in biological fluids and analyze p17 biological activity after binding to p17R, we investigated the conditions to maintain purified recombinant p17 preparations in a monomeric form. To this aim we performed gel-filtration experiments to evaluate if osmolarity of the solution could influence the p17 aggregation status. As shown in Figure 1A, the increase of ionic strength resulted in a drastic reduction of p17 oligomerization, with p17 in a monomeric form already at 0.6 M NaCl (upper panel). The retention volume of the p17 monomer is equal to 8.25 ml, according to that of myoglobin contained in the standard for calibration, which has a molecular weight of 17,000 Daltons. Lowering the ionic strength of the solution by dialysis against PBS (0.13 M), we observed a gradual process of p17 oligomers formation, with a volume of retention in gel-filtration of 5.36 ml (lower panel). Stability of these p17 oligomeric forms is extremely high since they are stable and detectable by Western blot under denaturating conditions. This finding allowed us to study the development of the p17 oligomerization process in solution under different NaCl concentrations (0.5, 0.2 and 0.1 M) by western blot and to establish the molecular weight of the resulting oligomers. Western blot analysis, performed using the monoclonal antibody (mAb) MBS-3 which recognizes the functional epitope AT20 (aa 9–28) in the p17 NH_2_-terminal region [18], [26], revealed that both monomeric and trimeric p17 are recognized by this mAb (Figure 1B). Under reducing conditions, p17 is mostly in monomeric form in a solution containing 0.5 M NaCl (lane a), whereas it is detected as an approximately 40.000 Daltons band in solutions containing lower NaCl concentrations, i.e., 0.2 M (lane b) and 0.1 M (lane c). A further confirmation of the dependence of p17 aggregation status by the ionic strength was obtained by ELISA, allowing the binding of GST-p17 to p17 as antigen on the solid-phase under different NaCl concentrations. As shown in Figure 1C, binding of GST-p17 – but not GST alone – to p17, as detected by an anti-GST mAb, increased concomitantly to the decreased NaCl concentration in the solution.

### Monomeric and trimeric p17 bind to p17R and show similar biological activity

**Figure 2:**
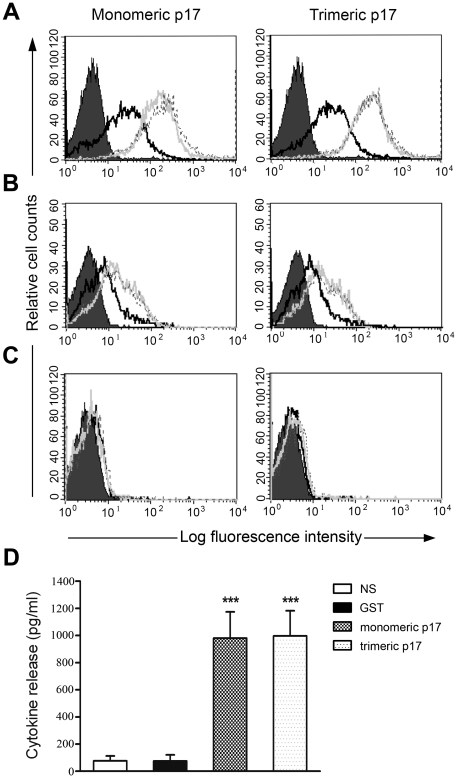
Binding of monomeric and trimeric p17 to p17R and induction of MCP-1 production. (A, B, C) Biotin-conjugated monomeric and trimeric p17 at 50 (black line), 200 (grey line) and 400 ng/ml (dotted line) was allowed to react with Raji (A), primary B cells (B) and H9 (C). Cells incubated with biotinylated GST were used as negative control (solid histogram). Binding of p17 to cells was detected by using APC-conjugated streptavidin. Data were analyzed using CELLQUEST Software and displayed as histograms. These results are representative of four different experiments with similar results. (D) Purified human monocytes were treated or not with GST, monomeric and trimeric p17 at a concentration of 1 µg/ml. Culture supernatants were collected 48 h after the stimulation of culture and analysed for the presence of MCP-1 by a standard quantitative ELISA. Bars represent the mean ± SD of four independent experiments performed in triplicate. Statistical analysis was performed by Wilcoxon matched pairs test. *** P<0.001, statistically different compared with GST.

We performed binding studies of monomeric and trimeric p17 expressed on Raji by flow cytometry. Binding of p17 to Raji cells was detectable at doses of proteins as low as 50 ng/ml (Figure 2A). However, saturation of p17Rs on Raji cells, evidenced by flow cytometry as the maximum increase in mean fluorescence intensity, was achieved with both monomeric and trimeric p17 at a dose ranging from 200 to 400 ng/ml (Figure 2A). Binding studies of monomeric and trimeric p17 to p17Rs expressed on human primary B cells confirmed the same results obtained with Raji cells (Figure 2B). No binding of monomeric and trimeric p17 to H9 cells, a human lymphoblastoid cell line lacking p17R, was observed at any dose tested (Figure 2C).

It is known that p17-treated human monocytes release MCP-1 in cell culture supernatant [20]. To assess whether monomeric and trimeric p17 preparations were able to induce MCP-1 production in human monocyte cultures, purified cells were grown in the presence or in the absence of the above p17 forms (1 µg/ml). GST was used in this experiment as negative control protein. Supernatants were collected at 48 h after stimulation and screened by ELISA for the quantification of MCP-1. Both monomeric and trimeric p17 preparations were able to induce similar peaks of MCP-1 production. Unstimulated cells – as well as GST-stimulated cells – released approximately 50 pg/ml of MCP-1 whereas a significant increase in the chemokine release was triggered by stimulation of cells with either monomeric (980 pg±193) or trimeric (996 pg±185) p17 preparations (Figure 2D). Statistical analysis of data showed no difference between MCP-1 production induced by monomeric and trimeric p17 preparations. This finding attests for the capability of p17 to exert biological activity independently of its aggregation status.

### The COOH-terminal region of p17 is not directly involved in p17R binding

**Figure 3:**
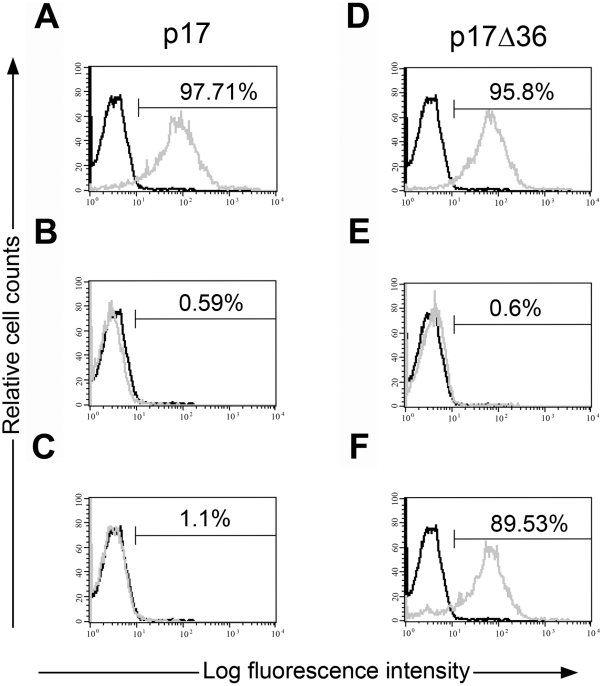
Inhibition of p17 and p17Δ36 binding to its cellular receptor p17R. Biotin-conjugated p17 and p17Δ36 were allowed to react with Raji cells in the absence (A, D) or in presence of anti-p17 mAbs MBS-3 (B, E) or MK-18 (C, F). The interaction of biotinylated-p17 and -p17Δ36 with p17R was detected in Raji cells by using APC-conjugated streptavidin (grey histogram). Cells incubated with biotinylated GST were used as negative control (black histogram). Data were analyzed by using CELLQUEST software and displayed as histograms. The percentage of cells, which interact with p17 and p17Δ36 cells is given in the upper right corner of each panel. These results are representative of four different experiments with similar results.

To assess whether different epitopes play a role in the interaction of p17 with its cell receptor, we took advantage of a set of anti-p17 mAbs developed in our laboratory. As expected, p17 binding to Raji cells (Figure 3A) was completely blocked by mAb MBS-3 (Figure 3B), which recognizes a linear epitope (aa 9–18) within the BH10-derived AT20 functional epitope [18], [26]. Other anti-p17 mAbs, and among them one named MK-1 [18], didn't show any neutralizing activity (data not shown). Surprisingly, mAb MK-18 showed a strong neutralizing activity (Figure 3C) despite its inability to recognize the AT20 peptide by solid-phase ELISA (data not shown). To identify the p17 functional region recognized by mAb MK-18 epitope mapping was performed with a series of decapeptides that completely spanned the p17 protein. The solid-phase peptides were individually screened for reactivity to mAb MK-18. Data obtained showed that the MK-18 binding region was in the p17 COOH-terminus and was included between aa 115 and 132 (data not shown). Comparison of NMR and X-ray structures of p17 [1], [21] evidenced a strong flexibility of its COOH-terminal region, in particular of the H5, that can come in close contact with the globular head. A structural contact between NH_2_- and COOH-terminal regions of p17 is confirmed by the development of a mAb recognizing a discontinuous epitope corresponding to aa 12–19 and aa 100–105 [27]. Therefore, the capability of displacing the binding of p17 to Raji cells by mAb MK-18 could be then attributed to the close proximity of the COOH-terminal region to the functional AT20 epitope located in the globular head of the viral protein and then ascribed to a phenomenon of steric hindrance. To better characterize the binding of p17 to Raji cells, we evaluated the capacity of a p17 protein lacking its fifth helix in the COOH-terminus to interact with cells. To generate the truncated form of the p17 protein we performed the deletion at the COOH-terminus of the BH10 p17 gene according to its secondary structure. A 96 aa long truncated form of p17 – named p17Δ36 – was produced and purified, whereas any attempt to purify other two COOH-terminal truncated forms of p17, the 90- (p17Δ42) and 85- (p17Δ47) aa long proteins was unsuccessful due to the predominance of hydrophobic stretches that turned these proteins into completely insoluble molecules. We have then evaluated the capability of p17Δ36 molecule to exert biological activity and shown that it was similar to p17 in its ability to bind p17R on Raji cells. Figure 3D shows specificity of binding of biotinylated p17Δ36 to p17Rs expressed on Raji cells since p17Δ36/Raji cell interaction was inhibited by mAb MBS-3 (Figure 3E) but not, as expected, by mAb MK-18 (Figure 3F).

### P17Δ36 induces a stronger and more prolonged AP-1 activation than full-length p17 in Raji cells

**Figure 4:**
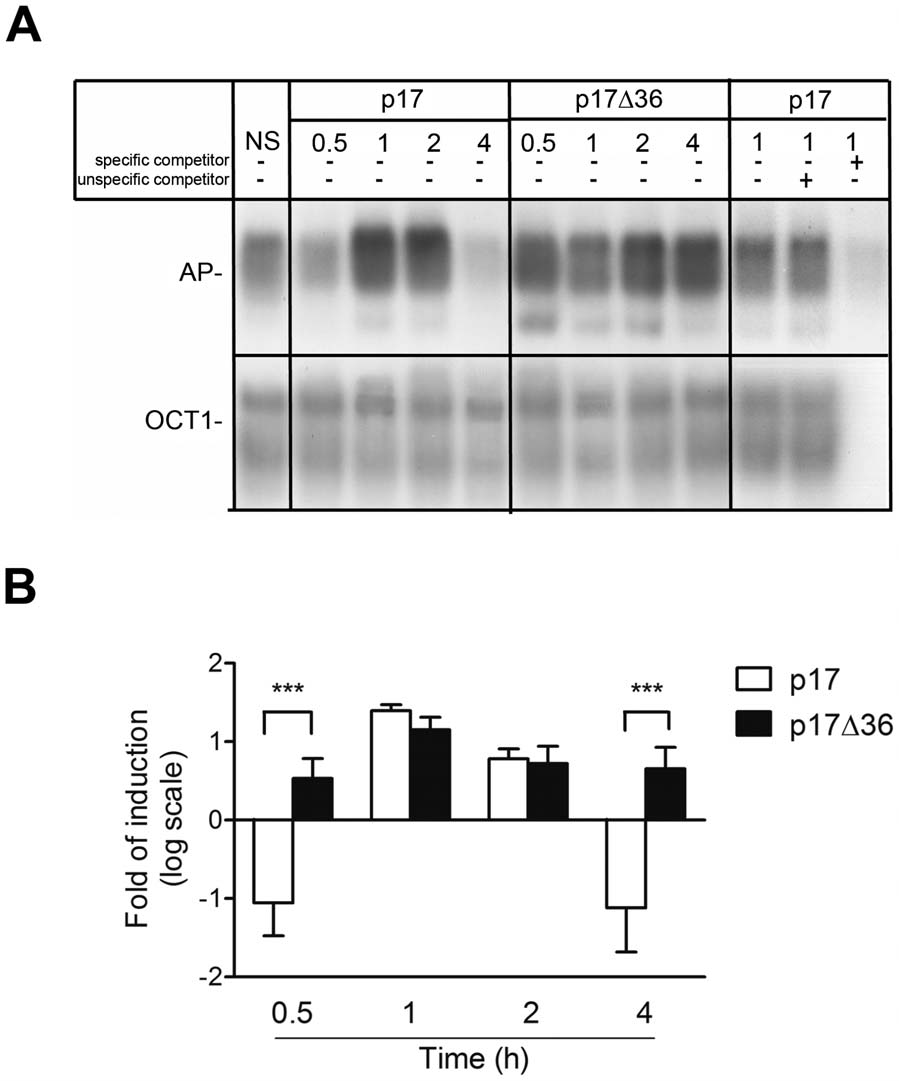
AP-1 activation in p17- and p17Δ36-treated Raji cells. (A, B) Nuclear extracts obtained from Raji cells collected after p17 and p17Δ36 treatments (1 µg/ml) were analysed for their binding activity to oligonucleotides specific for the transcription factor AP-1 and Oct-1. (A) Representative EMSA autoradiograms using nuclear extracts (10 µg per sample) from Raji cells collected at specific times 0.5, 1, 2 and 4 h after p17 and p17Δ36 treatment, and AP-1-specific radiolabeled oligonucleotides are shown. NS: p17-untreated cells. Protein-DNA complex specificity was confirmed by competition with an excess of unlabelled oligonucleotide probes. DNA-binding activity to Oct-1 was used as loading control. (B) The panel represents the densitometric analysis of changes in DNA-binding activity of AP-1 relative to Oct-1, expressed as fold of induction over p17-untreated cells. Ratios were expressed on a logarithmic scale in base 2. Bars represent the mean ± SD of four independent experiments. Statistical analysis was performed by two-way ANOVA. Bonferroni's post test was used to compare data: *** P<0.001.

Then we investigated if p17Δ36 retains the biological properties of the full-length p17. It has been recently shown that p17 activity is linked, at least in human primary monocytes, to the activation of the transcription factor AP-1 [20]. We verified whether p17 binding to Raji cells was followed by a similar increase in the AP-1 DNA-binding activity. Preliminary data obtained by EMSA showed that nuclear extracts from untreated Raji cells had a basal level of AP-1 DNA-binding activity and p17 activated AP-1 DNA-binding activity in a dose-dependent manner with a peak at 1 µg/ml dose (data not shown). Time-course analysis using 1 µg/ml of p17 showed that the activation of AP-1 DNA-binding activity is also time-dependent with a peak (2.6±0.26 fold increase) at 1 h after treatment and gradually decreased at 4 h after stimulation with p17 (Figure 4). The specificity of protein-DNA complexes was confirmed by competition experiments using an excess of specific unlabeled oligonucleotides. Moreover, pre-treatment of p17 with the neutralizing mAb MBS-3 blocked AP-1 activation (data not shown). Parallel experiments were run to assess the capability of p17Δ36 to activate AP-1. Similarly to p17, dose finding experiments established that optimal activation of AP-1 in Raji cells was achieved using p17Δ36 at the concentration of 1 µg/ml (data not shown). As shown in Figure 4B we found that p17Δ36 had a stronger and more prolonged AP-1 activation than the full-length counterpart. In fact, activation of AP-1 DNA-binding activity by p17Δ36 started already at 30 min (1.4±0.26 fold increase), with a peak (2.2±0.25 fold increase) at 1 h after treatment and persisted up to 4 h after stimulation (1.6±0.25 fold increase) (Figure 4B).

### P17 and p17Δ36 show a different pattern in modulating the Akt signalling pathway

The MAPK/ERK and PI3K/Akt pathways are the major intracellular signalling modules, which are known to regulate different cellular processes including cell proliferation, survival and malignant transformation [28], [29], and represent the upstream factors involved in AP-1 transcription factor activation [30], [31].

**Figure 5:**
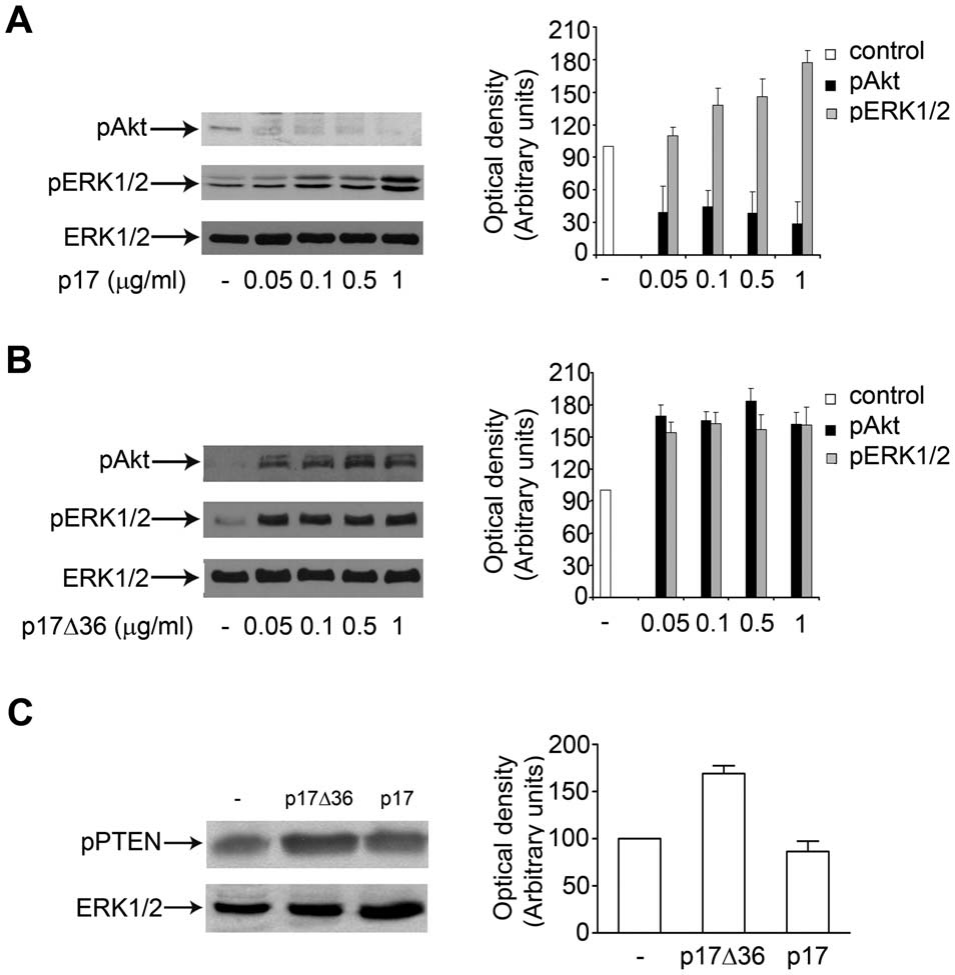
Effects of p17 and p17Δ36 stimulation on ERK1/2, Akt and PTEN activity in Raji cells. (A, B) Cells were treated for 5 min with 0.05, 0.1, 0.5 and 1 µg/ml of p17 (A) and 0.05, 0.1, 0.5 and 1 µg/ml of p17Δ36 (B). Untreated cells were used as control. Western blot analysis of Raji lysates shows that p17 inhibits the activation of Akt and induces the activation of ERK1/2 (A), as shown by the respective phosphorylation state at any concentration tested, verified by densitometric analysis and plotting of the pAkt/ERK1/2 and pERK1/2/ERK1/2. On the contrary, p17Δ36 induces the activation of Akt and ERK1/2 (B), as shown by the increased phosphorylation at any concentration, verified by densitometric analysis and plotting of the pAkt/ERK1/2 and pERK1/2/ERK1/2. In the left panel blots from one representative experiment of four with similar results are shown. In the right panels, values reported for phosphorylation of Akt and ERK1/2 are the mean ± SD of four independent experiments. (C) Cells were treated for 5 min with 1 µg/ml of p17Δ36 (lane 2) and p17 (lane 3). Untreated cells were used as control (lane 1). Western blot analysis of Raji lysates shows that p17Δ36 induces an increase of Ser/Thr pPTEN in contrast to p17, as verified by densitometric analysis and plotting of the pPTEN/ERK1/2. In the left panel one representative blot of four with similar results is shown. In the right panel values reported for Ser/Thr pPTEN are the mean ± SD of four independent experiments.

Since p17Δ36 shows a stronger and more prolonged AP-1 activation than the full length protein, we explored the capability of p17 and p17Δ36 to differently modulate the phosphorylation status of ERK1/2 and Akt. Raji cells stimulated for 5 min with the proteins showed activation of ERK1/2, as evidenced by the increase of ERK1/2 phosphorylation (Figure 5A and 5B). On the contrary, the two proteins showed a different phosphorylation pattern of Akt kinase: p17 significantly inhibited the activation of Akt as shown by the decreased phosphorylation state of the kinase (Figure 5A), whereas p17Δ36 induced a dose-dependent increase of Akt phosphorylation (Figure 5B). In conclusion, our data show that either p17 or p17Δ36 induce an analogous activation of ERK1/2 but show an opposite effect on the activation of Akt.

### P17 and p17Δ36 differentially regulate Akt phosphorylation by influencing PTEN activity

Down-modulation of PI3K/Akt pathway is known to be operated by PTEN, which antagonizes PI3K activity [25], [32], [33]. Therefore, the different effects of p17 and p17Δ36 on Akt phosphorylation may be dependent on the activation status of PTEN. The phosphorylation of PTEN regulates its own stability, activity and potentially its interaction with other proteins [34], [35], [36]. Ser/Thr phosphorylation has been implicated in the regulation of PTEN and there is a tight correlation between increased phosphorylation and PTEN inactivation [33], [37]. Therefore, we assessed the possibility that PTEN, after treatment with p17Δ36 or p17, could be present in Raji cells in a different phosphorylation status. As shown in Figure 5C, treatment of cells with p17Δ36 led to increased Ser/Thr phosphorylation of PTEN respect to unstimulated cells, while in contrast p17 stimulation reduced lightly the Ser/Thr phosphorylation level of PTEN. These data indicate that p17Δ36 inhibits PTEN activity by increasing Ser/Thr phosphorylation levels and, consequently, its ability to down-modulate Akt phosporylation. On the contrary, p17 treatment keeps PTEN in an active state, as attested by the low levels of pAkt. Therefore, the effects of p17 and p17Δ36 and on PI3K/Akt signalling have to be ascribed to a different modulation of PTEN activity.

### P17-induced activation of PTEN is mediated by ROCK

Recently, RhoA-associated kinase (ROCK), a downstream effector kinase of RhoA [38], has been reported to be involved in positive regulation of PTEN activity [22], [23], [24]. Indeed, ROCK can activate PTEN and target it to the plasma membrane, through an unknown mechanism, but most likely involving a physical interaction with PTEN, presumably by direct phosphorylation [22].

**Figure 6:**
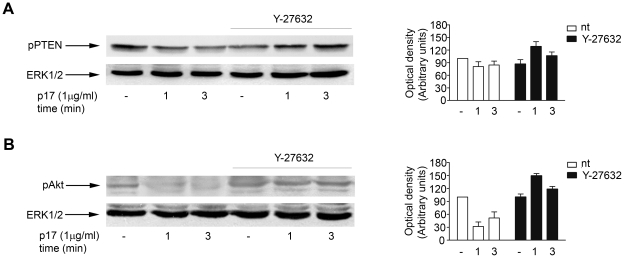
The effects of p17 are mediated by ROCK-induced activation of PTEN. Cells, pre-treated with Rock inhibitor Y-27632 (25 µM) for 20 min (lane 4, 5, 6), were stimulated for 1 and 3 min with p17 (1 µg/ml) (lane 5, 6). Cells not stimulated were used as negative control (lane 4). Cells not treated with inhibitor (lane 1, 2, 3) were also stimulated for 1 and 3 min with p17 (1 µg/ml) (lane 2, 3). Western blot analysis of Raji lysates shows that Y-27632 completely abrogates p17-induced decrease of both Ser/Thr pPTEN (A) and pAkt (B), as verified by densitometric analysis and plotting of pPTEN/ERK1/2 and pAkt/ERK1/2. In the left panels, blots from one representative experiment of four with similar results are shown. In the right panels, values reported for Ser/Thr pPTEN (A) and pAkt (B) are the mean ± SD of four independent experiments.

Since p17 is able to keep PTEN in an active state, we examined if p17 stimulation of Raji cells could activate PTEN through a pathway involving RhoA and ROCK. In particular, we determined whether pre-treatment with ROCK inhibitor Y-27632 [39], [40] was able to reverse the effects of p17 stimulation. The presence of Ser/Thr pPTEN and pAkt was thus assessed in the cytosolic extract of cells treated for 1 or 3 min with p17 in the presence or in the absence of Y-27632. The levels of Ser/Thr pPTEN and pAkt, which decrease in response to p17 stimulation (Figure 6), increased in cells pre-treated with ROCK inhibitor (Figure 6), similarly to cell treated with p17Δ36 only (Figure 5C and 5B), indicating inactivation and stabilization of PTEN in the cytoplasm. The finding that pharmacological inhibition of ROCK mimicked the same signalling of p17Δ36, identifies the RhoA/ROCK pathway as the major target of p17-mediated signalling, and establishes for the first time that p17 activates PTEN via its COOH-terminal tail.

### The HIV-1 p17 variant S75X shows a similar activity to p17Δ36 in modulating ERK1/2 and Akt signalling pathways in Raji and human primary B cells

**Figure 7:**
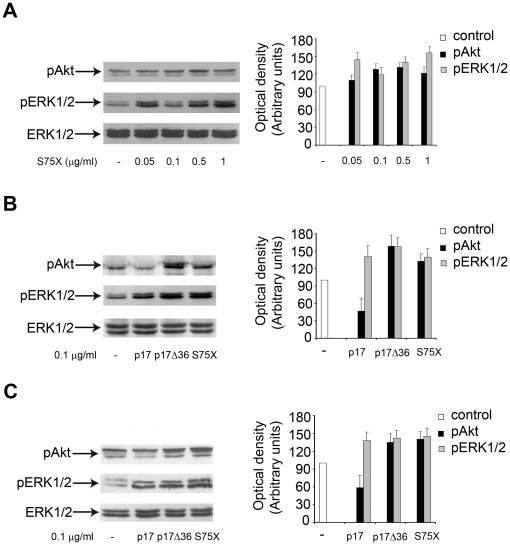
Effects of p17, p17Δ36 and S75X on pERK1/2 and pAkt in Raji and B cells. (A) Cells were treated for 5 min with S75X concentrations of 0.05, 0.1, 0.5 and 1 µg/ml. Untreated cells were used as control. Western blot analysis of Raji lysates shows that S75X activates Akt and ERK1/2, as shown by the increased phosphorylation of both kinases at any concentration tested, verified by densitometric analysis and plotting of the pAkt/ERK1/2 and pERK1/2/ERK1/2. In the left panel, blots from one representative experiment of four with similar results are shown. In the right panel values reported for phosphorylation of Akt and ERK1/2 are the mean ± SD of four independent experiments. (B, C) Raji (B) and human primary B cells (C) were treated for 5 min with 0.1 µg/ml of p17, p17Δ36 and S75X. Untreated cells were used as control. Western blot analysis of lysates shows that p17 inhibits the activation of Akt and induces the activation of ERK1/2 either in Raji or human primary B cells, as verified by densitometric analysis and plotting of the pAkt/ERK1/2 and pERK1/2/ERK1/2. On the contrary, p17Δ36 and S75X induce the activation of Akt and ERK1/2 at any concentration tested, verified by densitometric analysis and plotting of the pAkt/ERK1/2 and pERK1/2/ERK1/2. In the left panels blots from one representative experiment of three with similar results are shown. In the right panels values reported for pAkt and p ERK1/2 are the mean ± SD of three independent experiments.

We have recently described that different recombinant p17 proteins derived from Ugandan HIV-1 clade A and C isolates (S75X, S85X, S92X and S012X) are all capable of binding to p17R on Raji cells, despite differences in their primary aa sequence as compared to the BH10-derived protein [26]. Therefore we wondered if, upon their binding to p17R on Raji cells, S75X, S85X, S92X and S012X recombinant proteins trigger signalling pathways similar to the ones induced by p17Δ36. Stimulation of Raji cells for 5 min with different concentrations (ranging from 0.05 µg/ml to 1 µg/ml) of S85X, S92X and S012X variants induced signalling pathways which resembled that triggered by p17(data not shown). Interestingly, S75X induced an increase in the phosphorylation status of ERK1/2 and Akt at every dose tested (Figure 7A). A side by side comparison of signalling pathways triggered by p17, p17Δ36 and S75X (0.1 µg/ml) confirmed the capability of S75X to induce, similarly to p17Δ36, an increased phosphorylation of ERK1/2 and Akt (Figure 7B). These results highlight that one full length p17 variant, among the recombinant proteins we tested, displayed a p17Δ36-like activity despite the presence of the fifth helix H5 in the COOH-terminus of the protein. Taken together these data demonstrate that p17s derived from different HIV-1 isolates can differently impact on the signalling pathways leading to B cell proliferation.

These results were also confirmed using primary human B cells. Indeed, p17 was found to inhibit the activation of Akt, whereas p17Δ36 and S75X increased the phosphorylation status of Akt (Figure 7C).

### P17, p17Δ36 and S75X show different effects on colony formation

**Figure 8:**
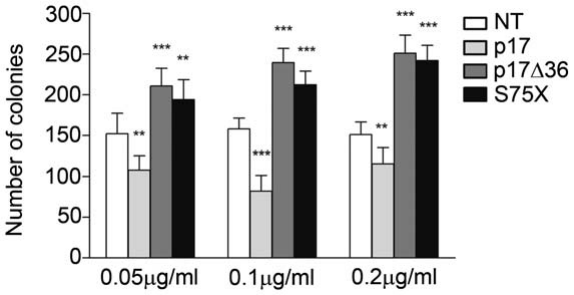
Effects of p17, p17Δ36 and S75X on colony-forming ability of Raji cell line. Cells were plated in six-well plates and, after two days, medium was replaced by fresh medium with various concentrations, 0.05, 0.1 and 0.2 µg/ml of p17, p17Δ36 and S75X. Cells not stimulated were used as negative control. The cell growth was analyzed by using MTT. Data represent the average number of colonies ± SD from three independent experiments performed in triplicate. The statistical significance between control and treated cultures was calculated using one-way ANOVA performed separately for each concentration of p17 variants, across the three groups. Bonferroni's post test was used to compare data: ** P<0.01, *** P<0.001.

Several studies have established a link between the PI3K/Akt pathway and human cancers [25], [41], [42]. To assess the capacity of p17Δ36 and S75X to induce cell growth and malignant transformation of B cells, compared to p17 one, we evaluated their influence on the capability of Raji cells to form colonies in soft agar. At any concentration tested (0.05, 0.1 and 0.2 µg/ml) p17 significantly inhibited the colony-forming ability of Raji cells compared to untreated control cultures (Figure 8). The colony number decreased from 152±25 to 107±17, from 158±13 to 82±19, from 151±15 to 115±19 with 0.05, 0.1 and 0.2 µg/ml of p17, respectively. On the contrary, p17Δ36 and S75X, used at the same concentrations, induced a significant increase of number of colonies compared to untreated cells (Figure 8). In fact, when cells were treated with p17Δ36 the colony number increased to 210±21 (0.05 µg/ml), to 239±17 (0.1 µg/ml), to 251±22 (0.2 µg/ml), and when S75X was used the colony number increased to 194±24 (0.05 µg/ml), to 212±16 (0.1 µg/ml), to 242±18 (0.2 µg/ml). This result demonstrates the selective anti-proliferative activity of p17 but, more interestingly, the capacity of p17Δ36 and of the natural variant S75X to increase cell growth and malignant transformation of Raji cells.

## Discussion

The HIV-1 matrix protein p17 has tendency to oligomerize, forming trimers of different crystal forms [21] but this occurs at high millimolar concentrations only. At nanomolar concentrations, as in the blood of HIV-1-infected individuals [12], p17 is present in monomeric form [21]. In our study, in order to reproduce conditions of p17/p17R interaction in biological fluids, we explored different conditions to obtain p17 protein preparations in a monomeric state and found that osmolarity of the solution influenced p17 aggregation. Data obtained showed that both monomeric and trimeric forms of p17 were able to interact with p17R and, as expected, to induce similar biological activities. This finding proves that p17 activity is independent from its aggregation form.

The functional p17 epitope involved in p17R binding was previously found to be located at the NH_2_-terminal region of the viral protein [18] and spans from aa 9 to 28, thus including a major portion of the polybasic region [26]. Indeed, an anti-p17 mAb (MBS-3), which recognizes this functional epitope on the native p17 protein, was found to block the p17/p17R interaction and, as a consequence, all p17 biological activities [18].

Using a monomeric p17 preparation we performed experiments aimed to study p17/p17R interaction in the presence of different p17 mAbs. Among many tested, mAb MK-18, a mAb recognizing an epitope in the COOH-terminal region located between aa 115 and 132, showed a potent p17 neutralizing activity. Thus, to better characterize the involvement of the p17 COOH-terminal region in the p17R binding, we evaluated the ability of a COOH-terminal truncated form of p17 (p17Δ36) to interact with p17R. Data obtained showed that p17Δ36 is a functional protein, being able to bind to p17R expressed on Raji cells. The use of the p17Δ36 protein has then unveiled that the binding of p17 to its cellular receptor does not involve the COOH-terminal region of the viral protein. Since NMR and X-ray analysis have evidenced a close proximity of the flexible H5 α-helix to the functional AT20 epitope on the globular head [1], [21] we hypothesize that the capability of mAb MK-18 to displace binding of p17 to p17R is likely due to mechanisms of steric hindrance. However, to better define a possible role of the H5 α-helix in the p17 biological activity, we investigated the signalling events triggered by p17 and p17Δ36 protein preparations in Raji cells.

We previously demonstrated that the MCP-1 expression stimulated by p17 in monocytes was primarily dependent on the activation of the transcriptional factor AP-1 [20], an indicator of external stimuli, as several signal transduction pathways converge to this molecule. However, the signalling pathways triggered by p17 have never been elucidated.

Here we show that following p17R interaction, p17Δ36 was capable of inducing a stronger and more prolonged AP-1 activation than the full length BH10-derived p17 protein. This finding was attesting for a functional p17 COOH-terminal region capable of triggering a signalling cascade which is distinct from the one evoked by the globular head. Therefore, we investigated the key signalling pathways, PI3K/Akt and MAPK/ERK, which are involved in AP-1 activation [30], [31] and play a pivotal role in basic cellular functions such as cell growth, survival, migration, angiogenesis and tumorigenesis [28], [29], [42]. Data obtained show that both p17 and p17Δ36 proteins induce the activation of ERK1/2, but they play opposite effects on the Akt signalling pathway. In particular, the Akt pathway was strongly activated in p17Δ36 stimulated cells, whereas it was inhibited in cells stimulated with p17. This finding allowed us to hypothesize that a NH_2_-terminal epitope of p17 may be responsible for an activation signal on PI3K/Akt signalling pathway opportunely balanced by a second inhibitory signal promoted by the COOH-terminal region.

Akt activation is known to require the formation of PIP_3_, produced by PI3K action, in order to be translocated to the cell membrane [43]. In normal mammalian cells the levels of PIP_3_ are tightly controlled by the combined effects of PI3K regulation and the action of phosphatases. PTEN is a lipid phosphatase, involved in regulation of cell growth, survival, invasion and tumor progression that inhibits PI3K-dependent activation of Akt by dephosphorylating PIP_3_ to PIP_2_ [32], [43], [44]. Inhibition of PTEN function results in increased PIP_3_ levels and subsequent Akt hyperactivation/phosphorylation [45], [46], [47]. PTEN possesses a COOH-terminal non catalytic regulatory domain that contains multiple putative phosphorylation sites [34], [48], which play an important role in its complex regulation and in the control of its biological activity [49]. Phosphorylation of the COOH-terminal region stabilizes PTEN protein in an inactive “closed” conformation, blocking the translocation of PTEN to the intracellular face of the plasma membrane [34], [50] and effectively inhibiting the dephosphorylation of the substrates of PTEN. The demonstration that p17Δ36 increases the Ser/Thr phosphorylation levels of PTEN indicates that it is capable of stabilizing the phosphatase in an inactivated phosphorylated closed state and, consequently, of increasing Akt phosphorylation. On the contrary, p17 treatment keeps PTEN phosphatase in a not phosphorylated active state. Therefore, we can conclude that the opposite effects of p17Δ36 and p17 on PI3K/Akt signalling are due to a different capability of the two proteins to modulate PTEN activity.

Recent studies have implicated the RhoA/ROCK pathway in the control of PTEN, whereby the Ser/Thr kinase ROCK, through an unknown mechanism, activates PTEN [22], [23]. In our experiments ROCK inhibitor Y-27632 [39], [40] completely abrogates p17 effects on PTEN and Akt phosphorylation, suggesting that the HIV-1 matrix protein, through its COOH-terminal region, down-modulates the PI3K/Akt cascade by activating PTEN via the RhoA/ROCK pathway.

**Figure 9:**
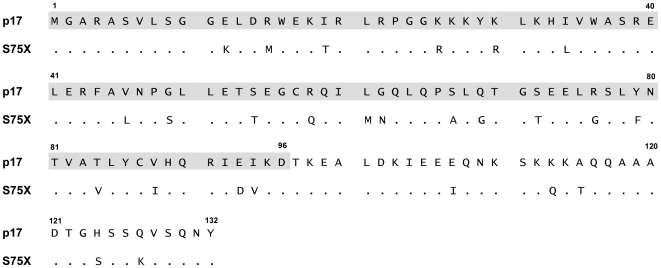
Alignment and comparison among p17, p17Δ36 and S75X aa sequences. Sequences are represented by the single-letter aa code. Each aa residue of Ugandan variant S75X not differing from p17 sequence is represented by a dot. P17Δ36 aa sequence is pointed out by a grey box.

There is no evidence, up to date, on the presence of COOH-terminal truncated forms of p17 in HIV-1 infected patients. The HIV-1 matrix protein p17 is well conserved across other retroviral proteins. However, we recently produced four recombinant p17 proteins derived from Ugandan HIV-1 clade A and C isolates, already identified in GenBank (S75X, S85X, S92X and S012X) containing major mutation points in their primary aa sequence, as compared to the BH10-derived protein [26]. Therefore, we have tested the hypothesis on the occurrence of a natural p17 variant with a biological activity similar to p17Δ36 in terms of ability to activate the Akt signalling pathway. Indeed, among p17s derived from Ugandan HIV-1 isolates, S75X was the only one found to up-regulate the PI3K/Akt signalling pathway. Interestingly, data obtained by soft agar growth assay show that S75X, like p17Δ36, possesses the capability of inducing cell growth and malignant transformation of B cells, whereas the BH10-derived p17 shows an antiproliferative activity according to its Akt signalling pathway. Therefore, specific mutations within the p17 primary aa sequence are required to generate signalling pathways critical for B cell proliferation and cancer insurgence. However, if we focus our attention to the entire protein sequences, we can observe that compared to the BH10-derived p17, the Ugandan variant S75X displays major substitutions that are not limited to the region that is truncated in p17Δ36, but scattered throughout the whole protein sequence (Figure 9). Conformational studies and development of site-specific mutagenesis on the BH10-derived p17 framework are needed to understand how mutations in the primary sequence of S75X are linked to PI3K/Akt signalling pathway activation, B cell growth and tumorigenesis.

Activation of the Akt signalling pathway is a hallmark of different types of malignancies and intensive studies on the PI3K/Akt pathway have firmly established a central role for Akt in tumorigenesis and cancer progression [25], [41], [42]. We know that patients with HIV-1 infection have a risk of developing B-cell lymphoma [51]. Consequently, a dysregulation of the PI3K/Akt signal transduction pathway in B cells, as shown for p17S75X, might contribute to the development of lymphoma in HIV-1-infected individuals.

The identification of p17 variants with possible oncogenic potential may represent not only a prognostic marker for the emergence of B-cell lymphoma in HIV-1-infected patients, but also a focal point for the development of effective anti-cancer therapies. In fact, the PI3K/Akt pathway might represent a promising therapeutic target [41], since significant progress has been made in developing small-molecule kinase inhibitors targeting the binding of pleckstrin homology (PH) domain of Akt to PIP_3_ or PIP_2_, which prevent Akt activation and membrane translocation [52], [53].

In conclusion, p17 exhibits a high degree of plasticity and diversity that allows formation of distinct ligand-receptor interactions capable of selectively activating or deactivating a variety of signalling pathways and appears to possess multiple, seemingly, conflicting targeting signals. The recently reported interest in drugs [54] and vaccines [55] specifically designed to block p17 function confirms the importance of this protein for HIV-1 infection and AIDS pathogenesis. Identification of new functional epitopes in the HIV-1 matrix protein, responsible of its extracellular actions, might be useful not only for a better and more detailed understanding of the molecular basis of its biological activities, but also for the development of new therapeutic strategies aimed to exogenously regulate p17 functions.

## Materials and Methods

### Cell cultures

Human lymphoblastoid cell lines Raji and H9 were obtained from the American Type Culture Collection (ATCC, Milano, Italy) and cultured in RPMI 1640 containing 10% fetal calf serum, 100 U/ml penicillin 50 µg/ml streptomycin and 1 mM L-glutamine. Before each experiment, cells were starved for 24 hours by serum deprivation (Phenol red-free RPMI containing 1% L-Glutamine).

### Human primary B cell preparation and cultures

Peripheral blood mononuclear cells (PBMCs) were freshly isolated from healthy donors, who gave informed consent to this research according to the Helsinki Declaration, by Ficoll gradient (GE Healthcare, Milano, Italy). B cell-enriched population was obtained from PBMCs by negative selection using human B cell Isolation kit from Miltenyi Biotec (Bologna, Italy). All of the above procedures were done under sterile conditions using reagents prepared in endotoxin-free water for clinical use. Purity of the resulting B cell population was assessed by staining with CD19-PE or PE-control IgG antibody (BD Biosciences, Buccinasco, Italy) for 30 minutes at 4°C and resulted 98% (±3%) CD19^+^. Viability of the purified cells was consistently higher than 98%. Freshly isolated B cells were resuspended at a density of 10^6^ cells/ml in prewarmed RPMI 1640 without FCS and then stimulated for 5 minutes at 37°C with recombinant p17, p17Δ36 and S75X at concentration of 0.1 µg/ml.

### Recombinant proteins

The coding sequence of HIV-1 matrix protein clade B isolate BH10 p17 (aa 1–132) [56] was amplified by Polymerase Chain Reaction (PCR) with specific primers that allowed us to clone the p17 sequence into the BamH1 site of the prokaryotic expression vector pGEX-2T (GE Healthcare). The coding sequences of HIV-1 matrix proteins derived from Ugandan clade A and C isolates (S75X, S85X, S92X and S012X) were amplified by PCR and cloned into BamH1 and EcoRI sites of the same vector as previously described [42]. P17Δ36, was cloned into BamHI and EcoRI sites of the same prokaryotic expression vector. The glutathione S-transferase (GST) fusion proteins were expressed in *Escherichia coli* and purified by using glutathione–agarose beads and thrombin, as described previously [18]. The recombinant proteins and GST were further purified (>98%) by reverse-phase Fast Performance Liquid Chromatography (FPLC). The absence of endotoxin contamination (<0.25 endotoxin U/mL) in protein preparations was assessed by Limulus amoebocyte assay (Associates of Cape cod Inc, Falmouth, MA). Purified p17 and p17Δ36 were also biotinylated by using AH-N-Hydroxysuccinimido-biotin (AH–NHS–biotin; SPA, Milan, Italy) according to the manufacturer's instructions.

### Dimensional analysis of p17 by gel-filtration

Experiments were performed using the AKTA purifier instrument (GE Healthcare) on Bioselect column (Bio-Rad Laboratories, Milano, Italy), by calibrating the instrument with a protein standard (Bio-Rad).

### Western blot analysis for identification of p17 monomers and trimers

Equal amounts of p17 protein in solution under different NaCl concentrations (0.5, 0.2 and 0.1 M) were resolved on a 12% SDS-polyacrylamide gel and then electroblotted onto a nitrocellulose membrane. The blots were incubated overnight at 4°C with mAb MBS-3 (purified in our laboratory), then the complex antigen-antibody was detected by incubation with peroxidase-coupled goat anti-mouse IgG (Thermo Scientific, Milano, Italy) for 1 h at room temperature and finally revealed using the ECL System (Santa Cruz Biotechnology, Heidelberg, Germany).

### Solid-phase ELISA under different NaCl concentration

Each well of 96-well microtiter plates (Maxisorp; Nunc, Roskilde, Denmark) was coated with 1 µg/ml of p17 in PBS (100 µl/well corresponding to 100 ng of protein) for 16 h at room temperature. Wells were then saturated with 200 µl/well of PBS containing 2% bovine serum albumin (BSA) for 1 h at 37°C. After washing four times with PBS containing 0.1% Tween-20, 100 µl of a solution at decreasing concentration of NaCl (1, 0.5, 0.2 and 0.1 M) of p17-GST (equal to 1 µg of p17-GST) was added to each well. The plates were incubated overnight at 4°C. The polymer formation between p17 adsorbed to solid-phase and p17-GST in solution, at different saline concentrations, was evaluated with a goat antibody anti-GST (GE Healthcare) and a secondary antibody horseradish peroxidase (HRP)-labeled anti-goat (DAKO, Milano, Italy). The signal was detected by adding tetramethylbenzidine substrate (TMB, Sigma) and colorimetric reaction was stopped with H_2_S 2N.

### Measurement of MCP-1

Supernatants of primary blood monocytes, purified as described previously [20], cultured for 48 h in the presence or absence of p17 at 1 µg/ml were assayed for the presence of monocyte chemoattractant protein (MCP)-1 using ELISA kits purchased from Endogen (Milano, Italy) according to the manufacturer's instructions. GST was also used in the experiment as negative control protein.

### Flow cytometry

Staining of cells for p17R expression was performed as previously described [18]. Briefly, the cells were incubated for 30 min on ice with biotin-conjugated recombinant p17 (monomeric or trimeric) at concentrations of 50, 200 and 400 ng/ml. After washing with PBS containing 1% FCS, they were incubated for 30 min on ice with allophycocyanin (APC)-conjugated streptavidin (BD Biosciences). All data obtained were analysed with CellQuest software (BD Biosciences).

### Binding and neutralization assays

Raji cells were incubated for 30 min on ice with different amounts of biotinylated p17 or p17Δ36, ranging from 50 to 400 ng/ml. Cells were then washed with cold PBS and further incubated for 30 min on ice with APC-conjugated streptavidin (BD Biosciences). Biotinylated-GST was used as negative control of the binding assay. Neutralizing anti-p17 mAb MBS-3, MK-1 and MK-18 were used in blocking assay. All data obtained were analyzed with CellQuest Software.

### Extraction of nuclear proteins

For each treatment condition, nuclear extracts of Raji cells were prepared from 1 or 2×10^7^ cells. The cells were washed with ice-cold PBS and lysed on ice for 10 min in 5× packed cell volume of lysis buffer containing 10 mM Hepes pH 7.9, 1.5 mM MgCl2, 10 mM KCl, 0.1 mM EGTA, 1 mM DTT, 0.2% Nonidet P-40 and protease inhibitor cocktail (Sigma-Aldrich). The nuclei were then pelleted by centrifugation at 3000 rpm at 4°C for 10 min and re-suspended in 3× packed nuclear volume of high-salt buffer containing 20 mM Hepes pH 7.9, 1 mM EDTA, 1 mM EGTA, 420 mM NaCl, 20% glycerol, 1 mM DTT and a protease inhibitor cocktail. The suspension was rocked for 20 min at 4°C, gently vortexed and subsequently centrifuged at 10000 rpm for 30 min at 4°C. The supernatant/nuclear extracts were collected and stored in aliquots at −80°C. The protein concentration was determined by using the Bio-Rad protein assay kit (Bio-Rad).

### Electrophoretic mobility shift assay (EMSA)

Nuclear extracts (10 µg of protein per sample), obtained as described above from Raji cells treated or not with p17 (0.05–1 µg/ml) and p17Δ36 (0.05–1 µg/ml), were incubated with 32P-labelled AP-1 probe [57], and the mobility of DNA–protein complexes (EMSA) was analysed as described previously [58]. Protease activity was stopped by the addition of ice-cold complete medium. For competition experiments, a 100-fold excess of specific unlabelled probe was incubated with nuclear extracts before addition of the radiolabeled probe. For loading controls, the nuclear extracts were analysed also for the DNA-binding activity of octamer-1 (Oct-1), whose site is present in many housekeeping genes [57]. Autoradiographic signals were quantified by Molecular Dynamics PhosphoImager (MDP) analysis (Typhoon 8600; Molecular Dynamics, Sunnyvale, CA).

### Western blot analysis

3×10^6^ cells were transferred into 10 cm dishes, treated with recombinant p17, S75X, S85X, S92X and S012X or p17Δ36 at different concentrations 0.05, 0.1, 0.5 and 1 µg/ml, then lysed in 200 µl of 10 mM Hepes (pH 7.9), 10 mM KCl, 1.5 mM MgCl_2_, 0.5 mM EGTA, 0.5 mM EDTA, 0.6% NP40, containing a mixture of protease inhibitors (Complete Mini Roche) and phosphatase inhibitors (sodium vanadate, PAO and sodium fluoride). When indicated, the cells were pre-treated at 37°C for 20 min with Y-27632 (25 µM). Equal amounts of total proteins were resolved on an 11% SDS-polyacrylamide gel and then electroblotted onto a nitrocellulose membrane. The blots were incubated overnight at 4°C with 1) mouse mAb pAkt (Santa Cruz Biotechnology), 2) mouse mAbs pERK1 and pERK2 (Santa Cruz Biotechnology), 3) rabbit polyclonal ERK antibody (Santa Cruz), 4) mouse mAb PTEN (Santa Cruz Biotechnology), 5) rabbit mAb pPTEN (Ser380/Thr382/383) (Cell Signaling Technology, Danvers, MA). The antigen-antibody complex was detected by incubation of the membranes for 1 h at room temperature with peroxidase-coupled goat anti-rabbit IgG or goat anti-mouse IgG (Thermo Scientific) and revealed using the ECL System.

### Soft Agar anchorage-independent growth assay

Raji cells (20000/well) were plated in 4 ml of 0.35% agarose, 5% charcoal-stripped FBS in phenol red-free RPMI 1640, with a 0.7% agarose base in six-well plates. Two days after plating, medium containing the proteins as indicated was added to the top of the layer and replaced every four days. After 10 days, 300 µl of 3-[4, 5-Dimethylthiazol-2-y1]-2, 5-diphenyltetrazolium bromide (MTT, Sigma) were added to each well and allowed to incubate at 37°C for 4 h. Plates were then placed in 4°C overnight and colonies >50 µm diameter were counted.

### Statistical analysis

Data obtained from multiple independent experiments are expressed as the mean ± SD. Data were analyzed for statistical significance using Wilcoxon matched pairs test, one-way and two-way ANOVA when appropriate. Bonferroni's post test was used to compare data. Differences were considered significant at P<0.05. Statistical tests were performed using GraphPad Prism v.5 Software.
